# TAp63α targeting of Lgr5 mediates colorectal cancer stem cell properties and sulforaphane inhibition

**DOI:** 10.1038/s41389-020-00273-z

**Published:** 2020-10-10

**Authors:** Yue Chen, Meng-huan Wang, Jian-yun Zhu, Chun-feng Xie, Xiao-ting Li, Jie-shu Wu, Shan-shan Geng, Hong-yu Han, Cai-yun Zhong

**Affiliations:** 1 Department of Fundamental and Community Nursing, School of Nursing, Nanjing Medical University, Nanjing, 211166 China; 2 Department of Nutrition and Food Safety, School of Public Health, Nanjing Medical University, Nanjing, 211166 China; 3 Suzhou Digestive Diseases and Nutrition Research Center, Suzhou Municipal Hospital, The Affiliated Suzhou Hospital of Nanjing Medical University, Suzhou, 215000 China; 4 Department of Clinical Nutrition, Sun Yat-sen University Cancer Center, State Key Laboratory of Oncology in South China, Collaborative Innovation Center for Cancer Medicine, Guangzhou, 510060 China; 5 Center for Global Health, School of Public Health, Nanjing Medical University, Nanjing, 211166 China

**Keywords:** Cancer stem cells, Cancer stem cells

## Abstract

Cancer stem cells (CSCs) have an established role in cancer progression and therapeutic resistance. The p63 proteins are important transcription factors which belong to the p53 family, but their function and mechanism in CSCs remain elusive. Here, we investigated the role of TAp63α in colorectal CSCs and the effects of sulforaphane on TAp63α. We found that TAp63α was upregulated in spheres with stem cell properties compared to the parental cells. Overexpression of TAp63α promoted self-renewal capacity and enhanced CSC markers expression in colorectal sphere-forming cells. Furthermore, we showed that TAp63α directly bound to the promoter region of Lgr5 to enhance its expression and activate its downstream β-catenin pathway. Functional experiments revealed that sulforaphane suppressed the stemness of colorectal CSCs both in vitro and in vivo. Upregulation of TAp63α attenuated the inhibitory effect of sulforaphane on colorectal CSCs, indicating the role of TAp63α in sulforaphane suppression of the stemness in colorectal cancer. The present study elucidated for the first time that TAp63α promoted CSCs through targeting Lgr5/β-catenin axis and participated in sulforaphane inhibition of the stem cell properties in colorectal cancer.

## Introduction

Colorectal cancer remains high incidence and mortality in recent years worldwide^1–3^. Despite the advances in early diagnostics and interventions, colorectal cancer frequently recurs and leads to heavy burden and death^4^. Studies have validated the crucial roles of cancer stem cells (CSCs) in the initiation, invasion, metastasis, and drug resistance in cancer progression^5^. Specific markers and several signal pathways have been found to participate in the development and maintenance of colorectal CSCs^6^. However, the critical targets and mechanisms which contribute to colorectal CSCs have yet to be fully elucidated.

The protein p63, including multiple protein isoforms, are important transcription factors belong to the p53 family. According to the transcription start sites, the p63 gene translates to the trans-activating (TA) isoforms that contain an N-terminal exon encoding a p53-like transactivation domain and ∆N isoforms without this domain. Alternative splicing events also generate at least three isoforms, α, β, γ^7^. The α isoform is known as the predominant isoform of p63 in epithelial cells^8^. Based on the unique structure of each isoform, the activities of them are always elusive in different diseases. Some studies have assessed the negative relationship between TAp63 and cancer progression^9,10^, which implies TAp63 may inhibit CSCs. Whereas it has been reported that p63 promoted the functions of embryonic stem cells^11,12^, and TAp63 also served to maintain adult stem cells^13^. These studies suggested the promoting role of TAp63 in stem cells. The conflicting effects of TAp63 in cancer and stem cells make it an interesting topic to reveal its functions in CSCs.

G-protein-coupled receptor 5 (Lgr5), a seven-transmembrane protein of the rhodopsin-like family, is recognized as a promoter of Wnt/β-catenin pathway. Upon R-Spondin (RSPO) ligand binding to the receptors, Lgr5 acts in cooperation with Wnt receptors to potentiate Wnt/β-catenin signaling^14^. The activation of Wnt/β-catenin pathway also leads to the expression of Lgr5, thus serving as a feedback loop to facilitate this pathway^15^. Further studies confirmed the relationship of Lgr5 and colorectal CSCs, thereby establishing Lgr5 as a marker of colorectal CSCs^16,17^. Although the different roles of p63 on Wnt/β-catenin response elements have been explored^18^, the interaction between p63 and Lgr5 is not well delineated.

Epidemiological and clinical studies have shown the effectiveness of cruciferous vegetables in cancer prevention and intervention^19,20^. Sulforaphane (SFN) is an isothiocyanate mainly obtained from cruciferous vegetables, which is reported to be a promising anticancer agent in daily food^21^. Previous studies have confirmed the suppressive effect of SFN on various CSCs, including breast, pancreatic, lung CSCs^22–24^. Here, we examined the efficacy of SFN on colorectal CSCs in both colorectal cancer cell lines and colorectal cancer xenograft. We also investigated the regulatory role of TAp63 and Lgr5 in colorectal CSCs and in the effects of SFN on colorectal CSCs.

## Results

### Enrichment of colorectal CSCs after sphere-forming

**Fig. 1:**
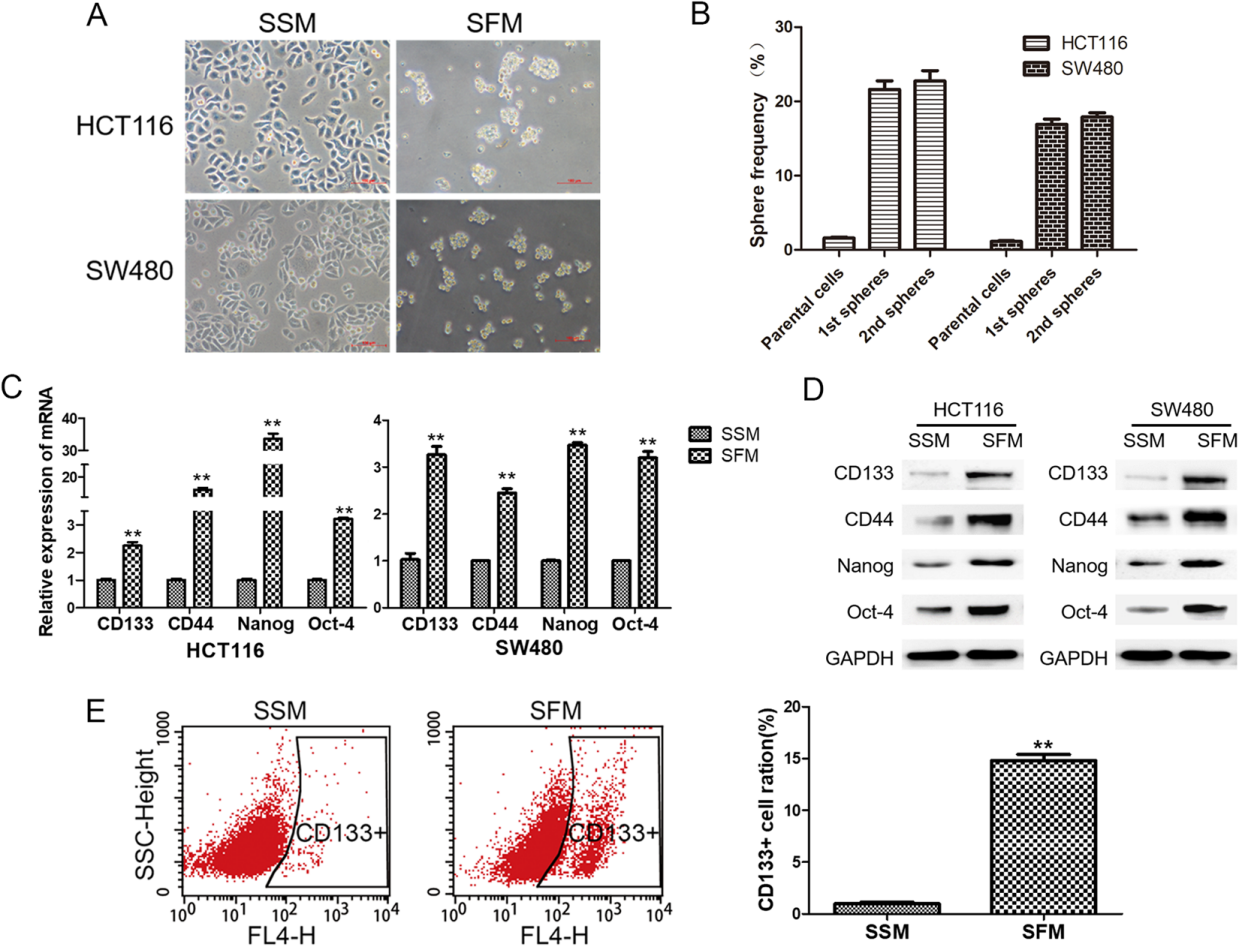
Enrichment of colorectal CSCs after sphere-forming. HCT116 and SW480 cells were maintained in SSM and SFM. **A** Images of cell morphology were obtained with a microscope. **B** Sphere frequency was calculated as the following formula: Sphere frequency = sphere number/seeded cell number. **C** qRT-PCR analysis for the mRNA levels of CD133, CD44, Nanog, and Oct-4. **D** Western blot analysis for the protein levels of CD133, CD44, Nanog, and Oct-4. **E** Flow cytometry analysis for CD133-positive cells. Data are expressed as mean ± SD, *n* = 3. **p < 0.01, compared with control group.

The sphere-forming assay has been widely used to enrich CSCs in vitro. Here, we examined the efficiency of the enrichment after serum-free medium (SFM) treatment. As shown in Fig. 1, spheres of HCT116 and SW480 cells were generated after SFM treatment, and the sphere frequency was significantly higher than the parental serum-sustained cells (Fig. 1A, B). The mRNA and protein expressions of colorectal CSC markers, including CD133, CD44, Nanog, and Oct-4, were significantly induced compared to the parental cells maintained in serum-sustained medium (SSM) (Fig. 1C, D). Flow cytometry analysis showed about 15% increase of CD133-positive cells (*p* < 0.05) after sphere-forming (Fig. 1E). These results suggested the characteristics of CSCs in HCT116 and SW480 sphere-forming cells cultured in SFM.

### TAp63α is associated with the stemness of colorectal CSCs

**Fig. 2:**
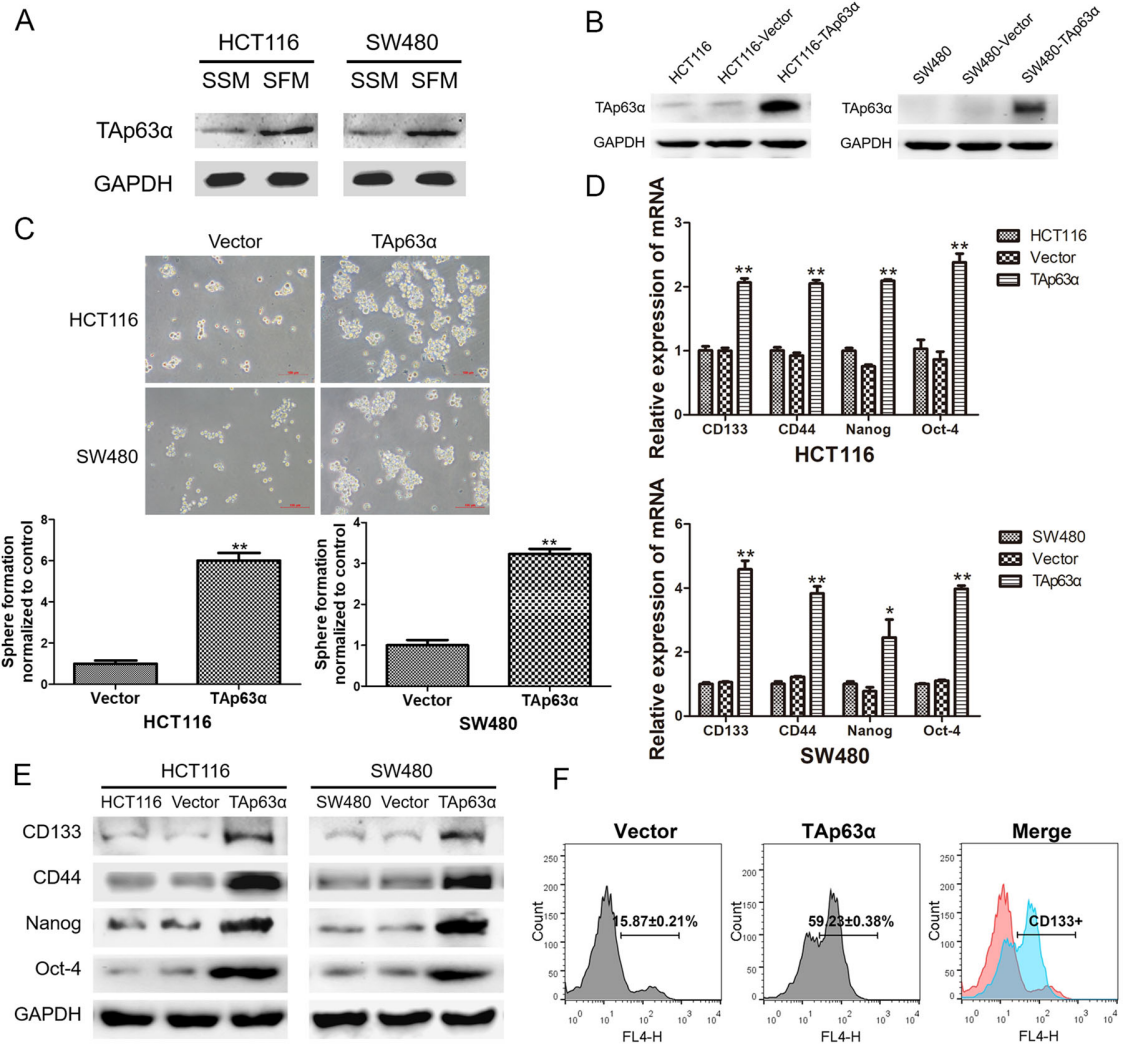
TAp63α is associated with the stemness of colorectal CSCs. HCT116 and SW480 cells were used to measure the function of TAp63α. **A** Western blot analysis for the protein levels of TAp63α after SSM and SFM treatment. **B** Western blot analysis for the transfection efficiency. **C** Sphere-forming assay to evaluate the self-renewal ability of the cells. **D** qRT-PCR analysis for the mRNA levels of CD133, CD44, Nanog, and Oct-4. **E** Western blot analysis for the protein levels of CD133, CD44, Nanog, and Oct-4. **F** Flow cytometry analysis for CD133-positive cells. Data are expressed as mean ± SD, *n* = 3. *p < 0.05 and **p < 0.01, compared with control group.

The preceding data prompted us to assess the expression of p63 in sphere-forming cells, and we found elevated expression levels of TAp63α in the spheres (Fig. 2A). To further determine the role of TAp63α in colorectal CSCs, we constructed a plasmid to elevate the expression of TAp63α in HCT116 and SW480 cells (Fig. 2B). The sphere-forming assay was applied to measure the self-renewal property of CSCs and showed that TAp63α facilitated the sphere formation ability of colorectal cancer cells. As shown in Fig. 2C, the size of the spheres was increased, and the number of the spheres was enhanced up to 6-fold (*p* < 0.05). We next examined the expression of mRNA and protein levels of colorectal CSC markers and found that overexpression of TAp63α upregulated the mRNA and protein levels of these markers (Fig. 2D, E). Flow cytometry analysis also showed that TAp63α transfection led to about 4-fold increase (*p* < 0.05) of CD133-positive cells compared to vector transfection group after sphere formation (Fig. 2F). These results suggested that TAp63α promoted the stem cell-like properties in colorectal cancer cells.

### TAp63α directly targets Lgr5 to promote stemness in colorectal CSCs

**Fig. 3:**
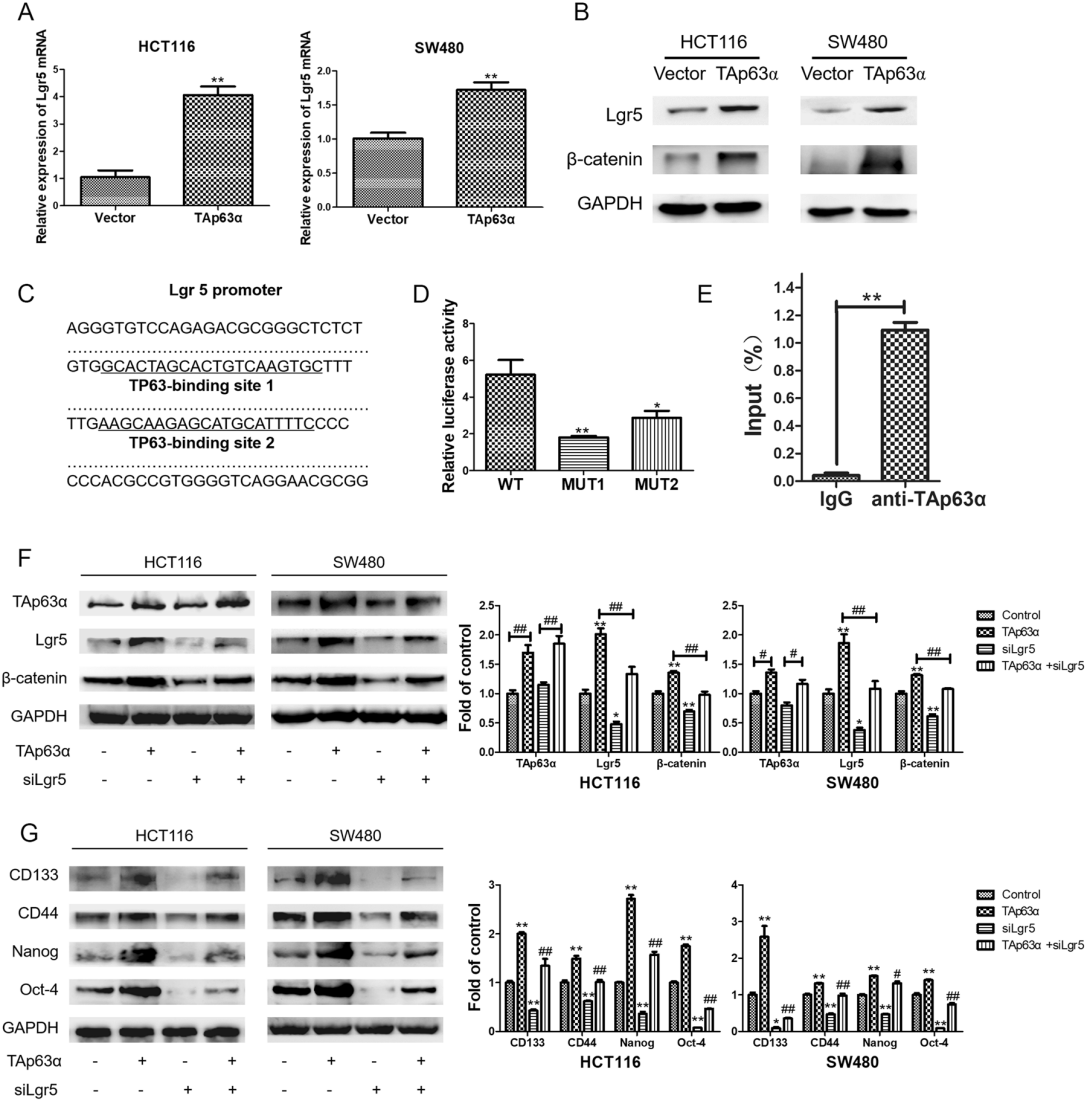
TAp63α directly targets Lgr5 to promote stemness in colorectal CSCs. HCT116 and SW480 cells were used to measure the regulation role of TAp63α on Lgr5. **A** qRT-PCR analysis for the mRNA levels of Lgr5 transfection. Data are expressed as mean ± SD, *n* = 3. **p < 0.01, compared with vector group. **B** Western blot analysis for the protein levels of Lgr5 and β-catenin. **C** Two binding sites of TP63 in the Lgr5 promoter region. **D** Dual-luciferase reporter assay for the transcriptional activity of Lgr5. Fold changes of the luciferase activity were relative to the vector group. Data are expressed as mean ± SD, *n* = 3. *p < 0.05 and **p < 0.01, compared with wild-type (WT) group. **E** ChIP assay for the binding capacity of TAp63α in Lgr5 promoter region after TAp63α was overexpressed. **F** Western blot and densitometric analyses for the protein levels of TAp63α, Lgr5, and β-catenin. Data are expressed as mean ± SD, *n* = 3. *p < 0.05, **p < 0.01, compared with control group. ^#^*p* < 0.05, ^##^*p* < 0.01. **G** Western blot and densitometric analyses for the protein levels of CD133, CD44, Nanog, and Oct-4. Data are expressed as mean ± SD, *n* = 3. *p < 0.05, **p < 0.01, compared with control group. ^#^*p* < 0.05, ^##^*p* < 0.01, compared with TAp63α group.

After TAp63α plasmid transfection, we observed upregulated mRNA and protein levels of Lgr5 in colorectal cancer cells (Fig. 3A, B). The immunoblotting analysis further confirmed the downstream β-catenin accumulation upon Lgr5 elevation (Fig. 3B). Bioinformatics method was then applied to show that there were two binding sites of TP63 in Lgr5 promoter region, and thus indicated the transcriptional regulation of TAp63α on Lgr5 (Fig. 3C). To identify the direct binding site of TAp63α, we constructed the wild-type luciferase reporter plasmid and the corresponding mutants. Dual-luciferase reporter assay showed that overexpression of TAp63α increased the transcriptional activity of Lgr5, while the luciferase activity was decreased with the mutation of the binding sites (Fig. 3D). Besides, ChIP assay confirmed the binding capacity of TAp63α in Lgr5 promoter region when TAp63α was overexpressed (Fig. 3E). Moreover, knockdown of Lgr5 with siRNA attenuated β-catenin accumulation and colorectal CSC markers expression triggered by TAp63α overexpression (Fig. 3F, G). Collectively, these findings illustrated that TAp63α directly targets Lgr5 at transcriptional level to promote colorectal CSCs.

### SFN diminishes CSC-like properties of colorectal cancer in vitro

**Fig. 4:**
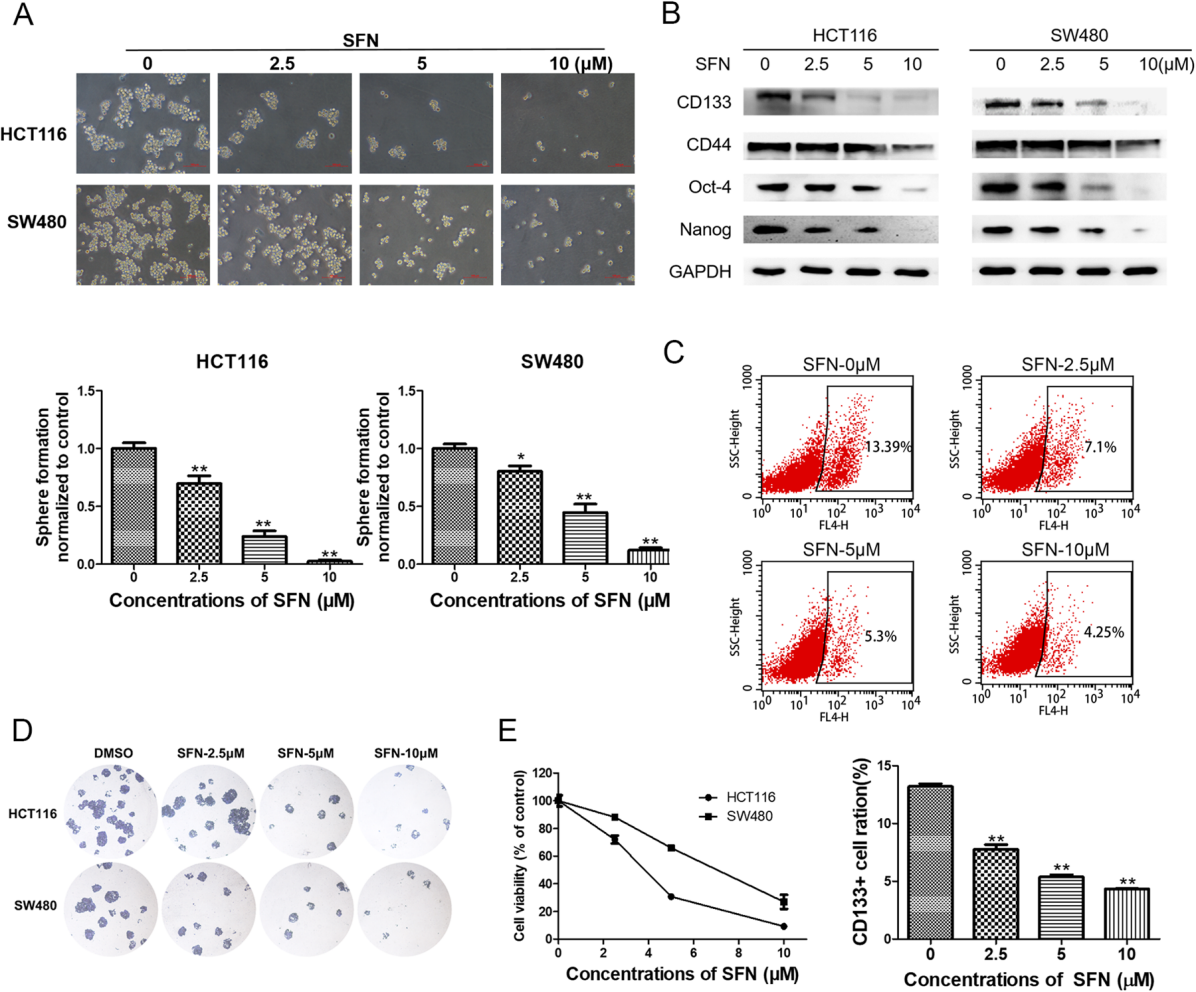
SFN diminishes CSC-like properties of colorectal cancer in vitro. HCT116 and SW480 spheres were treated with different concentrations of SFN for 4 days, and 0.1% DMSO was used as solvent control. **A** Sphere-forming assay was used to determine the size and number of spheres. **B** Western blot analysis for the protein levels of CD133, CD44, Nanog, and Oct-4. **C** Flow cytometry analysis for CD133-positive cells. **D** Colony formation of the spheres after SFN treatment. **E** Cell viability was measured by CCK-8 assay after SFN treatment of cell spheroids. Data are expressed as mean ± SD, *n* = 3. *p < 0.05 and **p < 0.01, compared with control group.

We then examined the effects of SFN on colorectal CSCs. As shown in Fig. 4, the size and the number of the colorectal cancer spheroids were decreased in a dose-dependent manner after the treatment of the spheroids with different concentrations of SFN for 4 days (Fig. 4A). Immunoblotting analysis showed that SFN treatment also led to the downregulation of the colorectal CSC markers, including CD133, CD44, Nanog, and Oct-4 (Fig. 4B). Besides, the percentage of CD133-positive cells was diminished from 13.39% to 4.25% in spheroids treated with 10 μM of SFN for 4 days (Fig. 4C). Furthermore, colony formation assay showed that SFN suppressed the formation of colonies in both cell spheroids (Fig. 4D), and CCK-8 assay confirmed the decline of cell viability in the spheroids (Fig. 4E). Overall, these results suggested that SFN inhibited colorectal CSC properties of HCT116 and SW480 cell spheroids in vitro.

### TAp63α mediates SFN-suppressed CSC-like properties

**Fig. 5:**
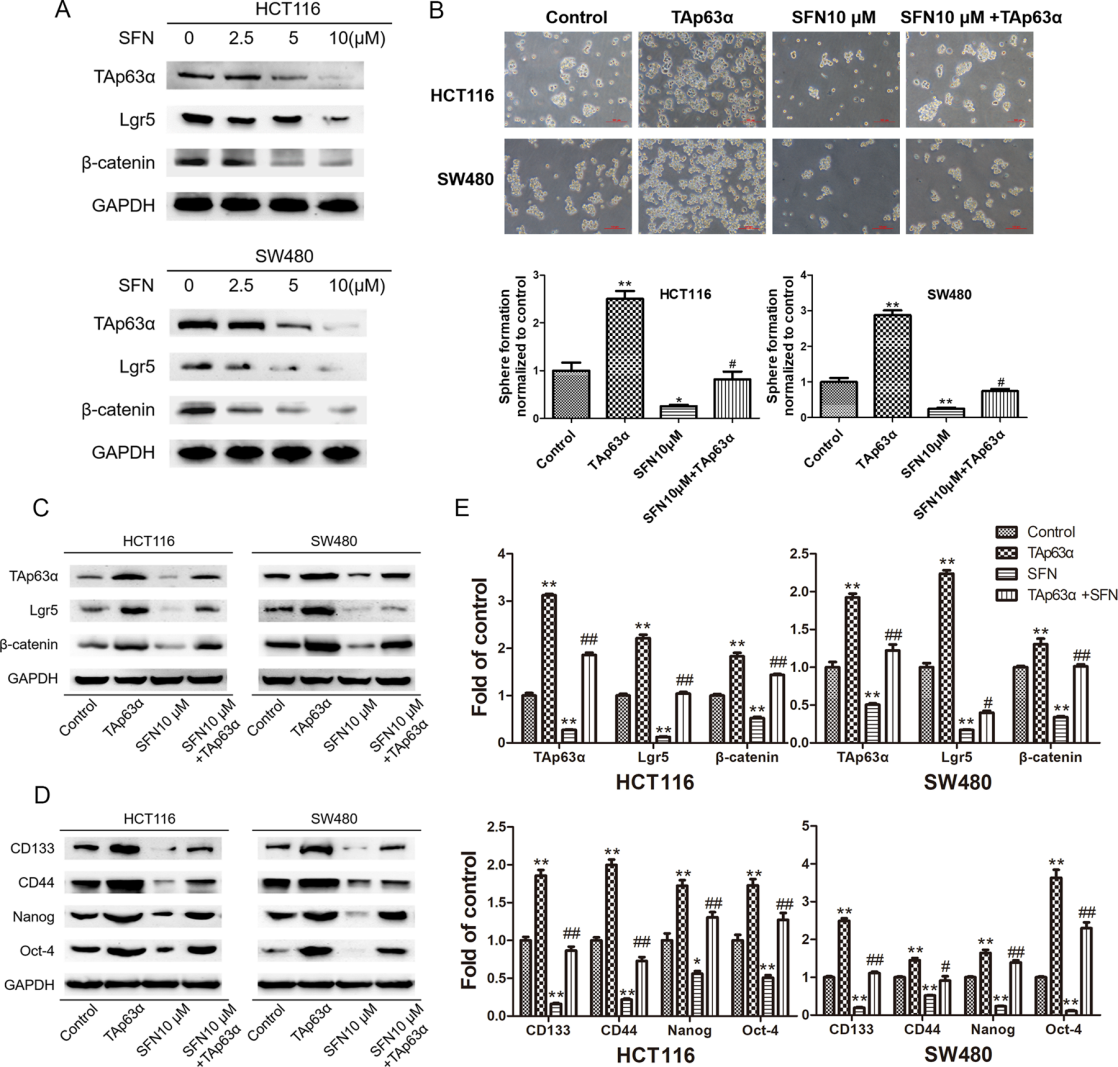
TAp63α mediates SFN-suppressed colorectal CSC-like properties. HCT116 and SW480 spheres were used to measure the role of TAp63α/Lgr5/β-catenin axis in SFN-suppressed colorectal CSCs. **A** Western blot analysis for the protein levels of TAp63α, Lgr5, and β-catenin. **B** Sphere-forming assay was used to determine the size and number of spheres. **C** Western blot analysis for the protein levels of TAp63α, Lgr5 and β-catenin. **D** Western blot analysis for the protein levels of CD133, CD44, Nanog, and Oct-4. **E** Densitometric analyses of western blotting. Data are expressed as mean ± SD, *n* = 3. *p < 0.05, **p < 0.01, compared with control group. ^#^*p* < 0.05, ^##^*p* < 0.01, compared with SFN group.

Considering the critical role of TAp63α/Lgr5/β-catenin axis in colorectal CSCs, we examined the effects of SFN on TAp63α and its downstream Lgr5 and β-catenin. As shown in Fig. 5A, concomitant with the repression of CSC properties, SFN treatment also led to downregulation of TAp63α in HCT116 and SW480 cell spheroids. Using sphere-forming assay, we verified that the inhibition of SFN on CSC self-renewal ability was reversed by TAp63α overexpression (Fig. 5B). Results of immunoblotting analysis further showed that SFN-induced Lgr5 and β-catenin suppression can be alleviated by TAp63α overexpression (Fig. 5C). Likewise, the repression of SFN on colorectal CSC markers was also diminished after TAp63α transfection (Fig. 5D, E). Together, these data suggested that TAp63α mediated the inhibitory effects of SFN on CSC properties in colorectal cancer.

### SFN inhibits TAp63α/Lgr5 axis and CSC-like properties in vivo

**Fig. 6:**
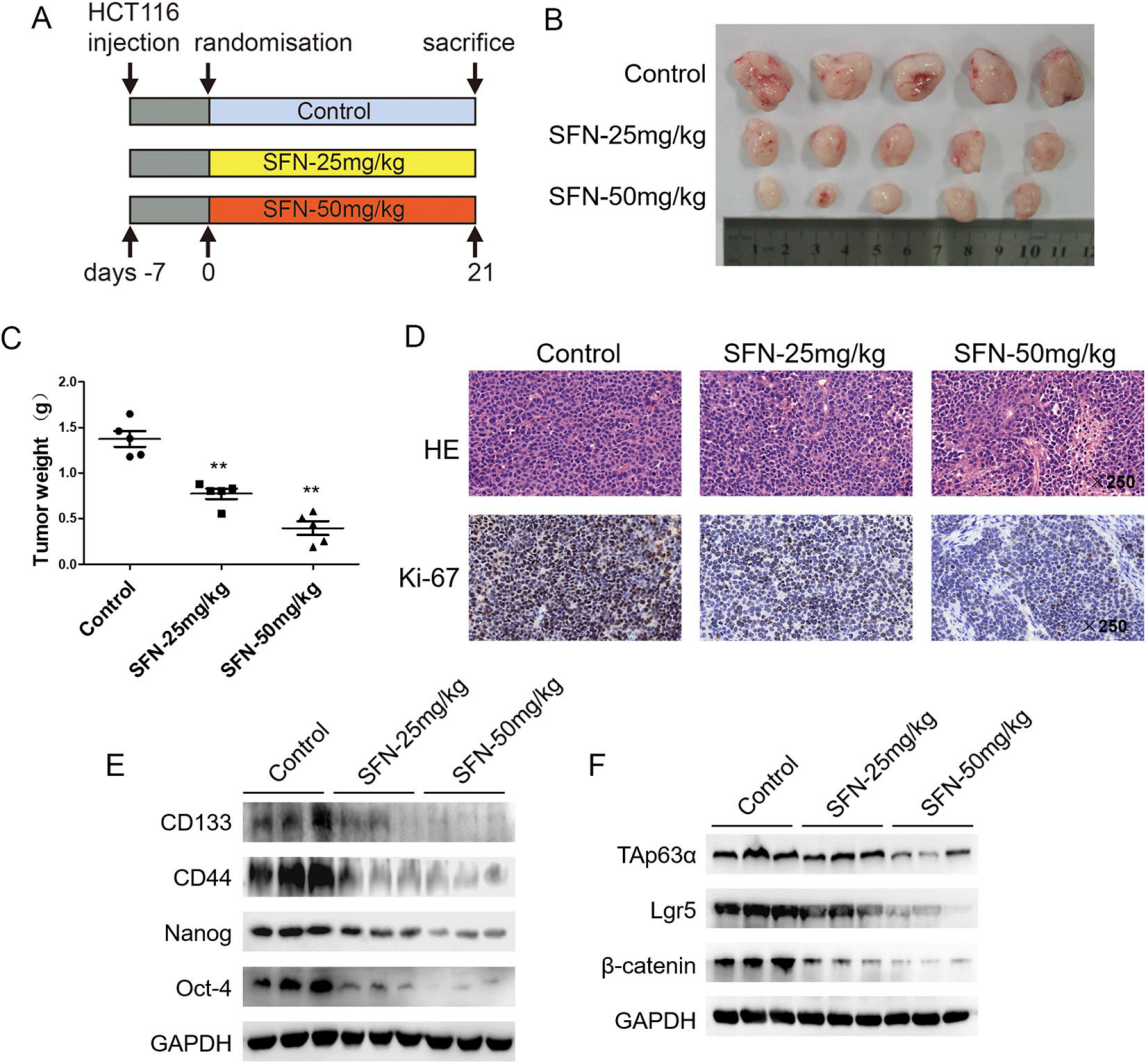
SFN inhibits TAp63α/Lgr5 axis and CSC-like properties in vivo. HCT116 cells were injected subcutaneously in the front dorsum of nude mice. **A** Mice were divided into three groups and treated with SFN or 0.9% sodium chloride solution. **B** Tumor volume after SFN treatment. **C** Tumor weight after SFN treatment. Data are expressed as mean ± SD, *n* = 3. **p < 0.01, compared with control group. **D** H&E and IHC staining for the xenograft. **E** Western blot analysis for the protein levels of CD133, CD44, Nanog, and Oct-4. **F** Western blot analysis for the protein levels of TAp63α, Lgr5, and β-catenin.

To investigate the effects of SFN on colorectal CSCs in vivo, HCT116 colorectal cancer xenograft model was established in nude mice. After injected subcutaneously with cells in the front dorsum, mice were divided into three groups and treated with SFN or 0.9% sodium chloride solution through intraperitoneal injection every 3 days (Fig. 6A). As shown in Fig. 6B, C, SFN treatment resulted in a significant decrease in tumor size and weight (*p* < 0.05). Immunohistochemistry staining validated the inhibition in tumor cell proliferation (Fig. 6D). As expected, the expression of colorectal CSC markers, including CD133, CD44, Nanog, and Oct-4, also decreased in the xenograft after SFN treatment (Fig. 6E). In concert with these colorectal CSC markers, inhibition of TAp63α/Lgr5/β-catenin axis was also observed (Fig. 6F). Collectively, these in vivo data regarding SFN effects on TAp63α activation and colorectal CSCs were consistent with in vitro results, suggesting the role of TAp63α/Lgr5/β-catenin axis in this process.

## Discussion

Several reports suggested the suppressive role of TAp63 in cancer progression. However, studies focused on stem cells showed that TAp63 promote the stem cell properties in embryonic and adult stem cells. Here, we investigated the function of TAp63α, one of the important p63 isoforms in epithelial cells, in colorectal CSCs, and showed that TAp63α directly targets Lgr5/β-catenin axis to promote stem cell properties in colorectal CSCs. Besides, we found that SFN inhibits colorectal CSC properties via TAp63α/Lgr5/β-catenin axis suppression.

The p63 family members are crucial regulators of cancer and have been found to play an important role in embryonic stem cells^25–27^. As reported by several studies, TAp63 suppressed the progression of cancers, and thus be considered as a promising strategy for therapeutics^9,10,28^. However, other studies have shown that TAp63 also promoted stem cell maintenance and differentiation^12,13^. Hence, the function of TAp63 in CSCs has attracted attention. The diverse isoforms of TAp63 may have different functions; while this study focused on α isoform because it is the predominant isoform of TAp63 in epithelial cells. We found that upregulation of TAp63α led to elevated self-renewal ability and CSC markers expression in colorectal cancer. These data indicated the activating role of TAp63α on colorectal CSCs, which support the previous studies in embryonic and adult stem cells, thus suggesting that TAp63α may be considered as a target of colorectal CSCs. To further explore the downstream genes of TAp63α in CSCs, we used bioinformatics approach and found two binding sites of TP63 on Lgr5 promoter. Results from dual-luciferase reporter and ChIP assay confirmed the existence and activity of the TAp63α binding complex. Moreover, the effects of TAp63α on Lgr5/β-catenin axis and colorectal CSC markers were also attenuated by Lgr5 silencing. Together, these results suggested that TAp63α directly targets Lgr5 to promote stemness in colorectal CSCs.

Since mounting evidence has established CSCs as the driving force in cancer progression, great efforts have been made to explore new strategies targeting CSCs. Wnt/β-catenin pathway is widely recognized as a crucial regulator of colorectal CSCs, and Lgr5 is a component of this pathway to potentiate Wnt signal and eventually leads to the activation of β-catenin. Besides, Lgr5 is now widely utilized as a stem cell marker in gastrointestinal cancers^29,30^, which indicates the role of Lgr5 in CSC properties. Here, we observed significant inhibition of CSC markers after Lgr5 silence, which suggested that Lgr5 may not only be used as a marker but also served as a target in colorectal CSCs.

In the past years, studies have reported the tumorigenesis role of ∆Np63, the critical ∆N isoforms of p63 expressed in epithelial tissues. It was shown that ∆Np63 promoted self-renewal and breast tumor growth through sonic hedgehog pathway in mammary CSCs^7^. Meanwhile, ∆Np63α was revealed as an oncogene for cooperating with Ras to promote tumor-initiating stem-like cells in skin stem cells^31^. These previous studies depicted the relationship between ∆Np63 and CSC properties. Although the present study focused on the role of TAp63α in colorectal CSCs, it is worth noting that further studies to ascertain the tumorigenic role of ∆Np63 in different cellular context, including colorectal CSCs, are necessary.

SFN is a natural product mainly obtained from cruciferous vegetables with anticancer activities^32^. Previous studies found that SFN exerted its anticancer effects through the regulation of drug-metabolizing enzymes^21,33^. Subsequent studies found that SFN also suppressed cell cycle and metastasis of cancer cells^20,34^. Recently, the inhibitory effects of SFN on CSCs have been reported^22–24^. However, the underlying targets by which SFN inhibited colorectal CSCs have yet to be fully elucidated. Here, we confirmed the inhibitory effects of SFN on colorectal CSCs both in vitro and in vivo, and found that SFN inhibited colorectal CSCs properties through TAp63α/Lgr5/β-catenin axis.

Although certain variables, such as the site of implantation, the properties of cell lines, and the growth properties of the xenograft, can affect the stability and reproducibility of xenograft formation^35,36^, cell line-derived xenograft models have been widely used in cancer research attribute to its high accessibility in laboratory settings^37^. Here, based on the results of our preliminary experiments, HCT116 cells were chosen to produce stable xenograft in mice in our system. It should be noted that although the in vitro results showed the promotion role of TAp63α on colorectal CSCs, future studies are warranted to explore the function of TAp63α in both in vitro and in vivo models.

In summary, we illustrated for the first time that TAp63α directly regulated Lgr5 expression to promote colorectal CSCs. Findings from this study suggested therapeutic potential of SFN in the treatment of colorectal cancer through inhibiting colorectal CSCs via targeting TAp63α/Lgr5/β-catenin axis.

## Materials and methods

### Cell culture and reagents

Human colon cancer cell lines HCT116 and SW480 were obtained from Shanghai Institute of Cell Biology, Chinese Academy of Sciences (Shanghai, China) and maintained in RPMI 1640 medium (Gibco, Carlsbad, CA, USA) supplemented with 10% fetal bovine (Gibco, Carlsbad, CA, USA), 100 units/mL penicillin and 100 mg/mL streptomycin (Gibco, Carlsbad, CA, USA) at 37 °C in incubator containing 5% CO_2_. SFN and dimethyl sulfoxide (DMSO) were purchased from Sigma (St. Louis, MO, USA).

### Sphere-forming assay

Cells were seeded in non-adherent dishes (Costar, NY, USA) in the incubator with 5% CO_2_ at 37 °C and maintained in serum-free medium (SFM) composed of DMEM-F12 (Gibco, Carlsbad, CA, USA), 20 ng/mL epidermal growth factor (Rocky Hill, NJ, USA), 10 ng/mL basic fibroblast growth factor (Rocky Hill, NJ, USA), 5 μg/mL insulin (Rocky Hill, NJ, USA), 0.4% BSA (Sigma, St. Louis, MO, USA), 2% B27 (Gibco, Carlsbad, CA, USA). The pictures and numbers of spheres were photographed under a microscope (Nikon, Tokyo, Japan) and counted if the diameter was greater than 50 μm. Colorectal cancer cells, 1st and 2nd spheres were seeded in 24-well plates (10,000 cells/well), and spheres were counted after 6 days. Sphere frequency was calculated as the following formula: Sphere frequency = sphere number/10,000. Spheroids viability was assessed by cell counting kit-8 (CCK-8) (Beyotime, Shanghai, China) according to the manufacturer’s instructions.

### Flow cytometry

For the detection of CD133-positive cells, cells were stained with CD133 antibody (Miltenyi Biotec, Teterow, Germany) at 4 °C for 10 min in the dark. Immunoglobulin G (IgG) isotype staining (Miltenyi Biotec, Teterow, Germany) was used as a negative control. Cell strainers were used to separate single cells before detection. The samples were analyzed using the FACS AriaIII system (Becton-Dickinson, San Jose, CA, USA) and the results were analyzed using FlowJo software (Ashland, OR, USA).

### Immunoblotting analysis

Immunoblotting analysis was performed as described previously^38^. In brief, 40 μg of total proteins in 15 μL were subject to 7.5–10% SDS-PAGE, transferred to nitrocellulose filter membrane (Millipore, Billerica, MA, USA), exposed to the antibodies, and detected by Chemiluminescence western blotting reagents (Cell Signaling Technology, Danvers, MA). GAPDH was served as the loading control. Antibodies used in the present study were purchased from Proteintech (Rocky Hill, NJ, USA).

### Quantitative real-time polymerase chain reaction (qRT-PCR)

Total RNA was isolated with Trizol reagent (Invitrogen, Carlsbad, CA, USA). The cDNA was synthesized with 5×All-In-One RT MasterMix (Applied Biosystems, Foster City, CA, USA). The EvaGreen 2×qPCR MasterMix (Applied Biosystems, Foster City, CA, USA) and LC96 real-time PCR system were applied for detection. The PCR primers were purchased from Beijing Genomics Institute (Beijing, China). Data were standardized by GAPDH. Primer sequences are listed as follows:

GAPDH-F, 5′-CAAGGTCACCATGACAACTTTG-3′;

GAPDH-R, 5′-GTCCACCACCCTGTTGCTGTAG-3′;

CD44-F, 5′-AGGATTTCCCCAGAACTTAG-3′;

CD44-R, 5′-ACAGGTCAAGATGGAAGATG-3′;

CD133-F, 5′-GCACTCTATACCAAAGCGTCAA-3′;

CD133-R, 5′-CTCCCATACTTCTTAGTTTCCTCA-3′;

Nanog-F, 5′-AGAAGGCCTCAGCACCTA-3′;

Nanog-R, 5′-GGCCTGATTGTTCCAGGATT-3′;

Oct4-F, 5′-ACATCAAAGCTCTGCAGAAAGAACT-3′;

Oct4-R, 5′-CTGAATACCTTCCCAAATAGAACCC-3′;

Lgr5-F, 5′-GAGTTACGTCTTGCGGGAAAC-3′;

Lgr5-R, 5′-TGGGTACGTGTCTTAGCTGATTA-3′.

### Transient transfection

HCT116 and SW480 cells were cultured in RPMI 1640 cell culture medium and seeded in six-well plates at a density of 2 × 10^5^ cells. Human pcDNA3.1-TAp63α and vector plasmids were purchased from Addgene (Rockville, USA). Human Lgr5 or control small-interfering RNA (siRNA) were purchased from RiBoBio (Guangzhou, China). Plasmids or siRNA were transfected into cells using the Lipofectamine 3000 reagent (Invitrogen, Carlsbad, CA, USA) following the manufacturer’s protocol. After transfections, cells were cultured in RPMI 1640 medium supplemented with 10% fetal bovine serum (Gibco, Carlsbad, CA, USA) before using for other experiments.

### Dual-luciferase reporter assay

The luciferase reporter plasmids contain the wild-type or mutant Lgr5 promoter into the pGL3 vector were generated by Genechem (Shanghai, China). Cells were seeded in 24-well plates and cultured 24 h before transfection. pGL3 vector, Lgr5-WT, Lgr5-MUT1 or Lgr5-MUT2 were co-transfected with pcDNA3.1-TAp63α and pRL-SV40 with lipofectamine 3000 reagent (Invitrogen, Carlsbad, CA, USA). Luciferase and Renilla activities were measured 48 h after transfection with the dual-luciferase reporter assay kit (Promega, Madison, WI, USA) following the manufacturer’s introductions. Renilla luciferase activities were used for normalization.

### Chromatin immunoprecipitation (ChIP)

ChIP assays were performed with chromatin immunoprecipitation kit purchased from Cell Signaling Technology (Danvers, MA, USA) in accordance with the manufacturer’s procedure. Anti-TAp63α and normal IgG antibodies were used to precipitate the DNA-protein complexes. Precipitated DNA was amplified with Lgr5 promoter-specific primers: 5′-TACTGATTGTGCGGAAAC-3′ (forward) and 5′-TGGAGAAAGTCGTCGAGT-3′ (reverse). The PCR products were analyzed by qRT-PCR analysis.

### Colony formation assay

Cell spheroids were separated into single cells and then seeded into cell culture dishes (Costar, NY, USA) maintained in RPMI 1640 medium at 37 °C in an incubator containing 5% CO_2_ for 10 days after treatment. Colonies were fixed in 4% paraformaldehyde solution for 10 min and stained with crystal violet for 10 min. After been washed with PBS, the colonies were then photographed and counted under a microscope (Olympus, Tokyo, Japan).

### In vivo experiments

For in vivo studies, female BALB/c nude mice (4 weeks old, 20 g) were purchased from Shanghai Animal Laboratory Center and maintained in the Experimental Animal Center at Nanjing Medical University with appropriate sterile filter-capped cages. All animal experiments were conducted under the instructions of the Guide for the Care and Use of Laboratory Animals of the National Institutes of Health strictly. The protocol was approved by the Committee on the Ethics of Animal Experiments of Nanjing Medical University (IACUC-1802010). HCT116 cells (5 × 10^6^ each) were injected subcutaneously in the front dorsum. At 7 days post-transplantation, the xenografts were randomized into three groups (five mice in each group). SFN or 0.9% sodium chloride solution were given through intraperitoneal injection every 3 days. No blinding was conducted during the experiments. The tumor volumes were calculated as the following formula: volume (mm^3^) = length × width × width/2. After 4 weeks of treatment, the mice were sacrificed, and the xenografts were removed for examination.

### Tissue immunohistochemistry staining

Tumor xenografts were fixed in 4% paraformaldehyde solution before paraffin embedding. Hematoxylin and eosin (H&E) and IHC staining were performed by the Servicebio (Wuhan, China). Briefly, paraffin-embedded sections were deparaffinized and hydrated in xylene, ethanol, and water. Sections were incubated with primary antibody at 4 °C overnight, followed by the corresponding secondary antibodies and developed with diaminobenzidine. The slides were counterstained with haematoxylin and mounted in xylene mounting medium for examination. Pannoramic scanner system was used to observe the cell images.

### Statistical analysis

All data are expressed as mean ± SD. Student’s *t* test was used to compare the difference between two groups. One-way analysis of variance (ANOVA) with Dunnet or Bonferroni post hoc test was used for multiple comparisons. Significant difference was taken as *p < 0.05 or **p < 0.01 or #*p* < 0.05. All analyses were performed with SPSS version 11.0 software.
